# Current State of LGBTQIA+ Health Education in Low- and Middle-Income Countries: A Narrative Review of Educational Gaps in Transgender, Intersex/Differences of Sex Development (DSD), and Sexual and Gender Minority Healthcare

**DOI:** 10.7759/cureus.113111

**Published:** 2026-07-21

**Authors:** Abdullah Aslam, Nusrat Fatima, Arzeen Khan, Maazan Mubasher, Tahmeed Ul Haq, Mohammad Jahanzaib, Mahad A Khan, Arham Amir Khawaja, Hasan Saeed, Haseeb Mehmood Qadri

**Affiliations:** 1 Medical College, Aga Khan University, Karachi, PAK; 2 Respiratory Medicine, Portsmouth Hospitals University NHS Trust, Portsmouth, GBR; 3 Medical School, Imperial College London, London, GBR; 4 Medical School, Lahore Medical and Dental College, Lahore, PAK; 5 Medical School, Ayub Medical College, Abbottabad, PAK; 6 Internal Medicine, Allama Iqbal Medical College, Lahore, PAK; 7 Internal Medicine, Islamabad Medical and Dental College, Islamabad, PAK; 8 General Surgery and Surgical Oncology, Shaikh Zayed Medical Complex, Lahore, PAK; 9 Histopathology, Shifa International Hospital, Islamabad, PAK; 10 General Surgery, Lahore General Hospital, Lahore, PAK; 11 Neurological Surgery, Punjab Institute of Neurosciences, Lahore, PAK

**Keywords:** curriculum, education, gay, health care, health personnel, lesbian, lgbtq+, low- and middle-income country

## Abstract

Sexual and gender minorities, LGBTQIA+ individuals, transgender individuals, and intersex individuals represent a small proportion of the population and encompass diverse sexual preferences, gender identities, sexual characteristics, and gender expressions. These individuals often present with unique health needs due to biological differences in anatomy, physiology, or genetic sexual characteristics. Despite these differences, education regarding their healthcare is insufficient in medical curricula worldwide, specifically in low- and middle-income countries (LMICs). Medical students are often unaware of the specific needs of these individuals and, hence, show a willingness to learn more so that they are better equipped clinically. Health care providers (HCPs) often lack the required knowledge and may behave unprofessionally toward these patients. This may include marginalisation, discrimination, and non-consensual examinations. Such instances make these individuals less likely to seek medical care. Thus, there is a dire need to integrate content about their healthcare needs into medical education and clinical practice in LMICs to prevent unprofessional conduct and improve health outcomes. This review highlights the existing literature on the educational and clinical preparedness of medical students and HCPs related to the healthcare needs of the LGBTQIA+ population, with a specific focus on intersex/differences of sex development (DSD) individuals, and accordingly puts forward recommendations for improvement.

## Introduction and background

Sexual orientation, gender identity, gender expression, and variations in sex characteristics are distinct but overlapping domains within lesbian, gay, bisexual, transgender, queer, intersex, and asexual (LGBTQIA+) health. Lesbian, gay, and bisexual identities describe sexual orientations, while transgender identities relate to gender identity. Intersex refers to a range of conditions in which a person's sex characteristics do not fit typical binary definitions of male or female. In the context of medical education, intersex is commonly described as a congenital variation in anatomical, physiological, or genetic sex characteristics. The condition of being intersex is also known as differences of sexual development (DSD) [1]. Reported prevalence estimates for intersex populations vary depending on the definitions, diagnostic criteria, and methodological approaches used across studies. Clinical presentations of individuals with DSD are mostly described through care pathways and lived experiences. Atypical genital appearance at birth can lead to repeated examinations, while some cases are recognised later at puberty [2].

Early recognition of intersex/DSD is important because key care decisions are often made at an early stage of a child's development [2]. Multidisciplinary collaboration is therefore essential to ensure competent, inclusive, and ethical medical care [3].

Physicians play a crucial role in the diagnosis and long-term care of individuals with DSD. They are expected to take sensitive histories, perform accurate physical examinations, make evidence-based decisions about care options, and counsel patients and families [4]. Clinicians should also support long-term follow-up across the life course and ensure safe access to healthcare. Therefore, undergraduate training related to DSD is important. However, many medical schools still allocate limited time to teaching these conditions. A study found that the median time spent on LGBTQIA+ topics in medical schools in the United States and Canada was five hours. In Australia and New Zealand, 0-5 hours were dedicated to teaching LGBTQIA+ content during the required pre-clinical phase [5,6]. This limited coverage leaves students with knowledge gaps and uncertainty about addressing the health concerns of transgender patients. High-Income Countries (HICs) generally have more formal training opportunities and infrastructure, but coverage remains inconsistent [1]. Some HIC institutions also run multidisciplinary clinical electives with placements across services [7]. In contrast, evidence from LMICs indicates under-resourced curricula and persistent learner knowledge gaps despite a strong willingness to learn [8].

There are significant gaps in the literature on LGBTQIA+ health needs and outcomes in low- and middle-income countries (LMICs). To address these, this narrative review included studies showing a persistent mismatch between the complex, lifelong care needs of intersex, DSD, and wider Sexual and Gender Minority (SGM) patients and how prepared clinicians feel to meet them. Reviews of medical education note that teaching in this area has traditionally been limited and remains difficult to implement because of ongoing barriers. The case is especially strong in LMIC settings, where the literature is scattered and not well synthesised. This review synthesises data from LMICs and clarifies the priority competencies of recognition, counselling, referral, and multidisciplinary follow-up. In addition, it identifies feasible, context-appropriate training strategies to reduce harm and improve patient-centred care for individuals with DSD.

## Review

Methodology

A comprehensive search was conducted in February 2026 using PubMed as the primary database for this narrative literature review, in addition to a manual search for relevant articles across Google Scholar. Terms including "LGBTQIA+", "DSD", "Undergraduate Medical Education (UGME)", and "LMICs" were used in varying combinations to formulate search strings. To further broaden the search, LMIC names were sequentially incorporated into the search strings. Low- and middle-income countries were determined according to the 2025 World Bank classification of countries by income level. Articles published in English over the last 10 years were considered. Relevant peer-reviewed articles, reviews, and policy documents focusing on undergraduate medical education and clinical preparedness were included and subsequently analysed. They were stratified on the basis of region, study design, and outcomes. Due to the limited availability of peer-reviewed studies on LGBTQIA+, intersex, DSD, and transgender health in LMICs, relevant grey literature was included to supplement the evidence.

The primary objective was to evaluate the current state of knowledge and clinical preparedness of medical students and HCPs in LMICs for caring for LGBTQIA+ individuals, with a specific focus on intersex/DSD individuals. The secondary objective was to identify and report feasible suggestions or interventions to address these gaps in the provision of care to the LGBTQIA+ community in LMICs.

Results

A systemic gap in LGBTQIA+ healthcare education and preparedness is consistently evident in the literature from various LMICs across South Asia, Africa, and the Middle East. Studies show that medical students and healthcare providers lack formal training, yet express a willingness to learn. Analysis of curricula from India demonstrates that sexual and gender minorities are pathologised or omitted completely from the curriculum. Such revisions increase the risk of further regression. More severe consequences are highlighted in patient-reported qualitative evidence from LMICs. These include the absence of care protocols, non-consensual medical interventions, discrimination, and privacy violations. Commentaries, particularly from Africa and Afghanistan, show that stigma associated with intersex conditions is ingrained in sociocultural norms and institutional systems, and this directly discourages healthcare-seeking behaviours (Table 1).

Overall, the studies highlight three major takeaways: first, educational omission and pathologisation directly lead to healthcare inequity; second, trainees are willing to learn; and third, structured curriculum integration and staff training are repeatedly recommended as necessary interventions. This evaluation of the LMIC landscape reflects not resistance to reform, but a gap between policy intent, curricular implementation, and clinical practice (Table 1). 

**Table 1:** Summary of the included literature. Alim et al. 2022 [2], Martins et al. 2020 [8], Ahmer et al. 2021 [9], Humayun et al. 2025 [10], Keles et al. 2018 [11], Isano et al. 2023 [12], Ehlers et al. 2001 [13], Muller et al. 2017 [14], Khalaf et al. 2022 [15], Torres et al. 2024 [16], Khatoun et al. 2025 [17], Agarwal et al. 2022 [18], Razai et al. 2023 [19], Omohwovo et al. 2026 [20], Hindus for Human Rights [21], Healthcare: Difficulties Of LGBTQ Refugees From The Middle East And North Africa [22].

Reference	Objective	Study Type and Country	Key Finding/Major Takeaway/Notes
Empirical studies			
Ahmer et al. 2021 [9]	To determine the knowledge and attitudes of medical students and HCPs regarding intersex and transgender individuals in Karachi.	Cross-sectional study; Pakistan	Medical Students and HCPs are aware of intersex and transgender; several misconceptions about intersex and transgender students are willing to learn
Humayun et al. 2025 [10]	To explore the educational and healthcare barriers faced by the ‘Hijra’ (Transgender) population and understand their experiences and perceptions regarding discrimination, access to education, and healthcare services	Exploratory qualitative study; Pakistan	Transgenders face barriers in accessing education and healthcare, including discrimination, neglect, and derogatory treatment from HCPs
Keles et al. 2018 [11]	To assess how LGBT+ individuals evaluate their experiences when receiving healthcare services.	Cross-sectional qualitative study; Turkey	LGBT+ individuals are exposed to discrimination, prejudice, and maltreatment in healthcare settings
Martins et al. 2020 [8]	To assess knowledge, attitude, and perceived need for transgender education in medical students.	Cross-sectional study; Pakistan	Students report insufficient education but positive attitudes; strong perceived need for inclusion into the curriculum
Isano et al. 2023 [12]	To explore the personal experiences of transgender women and men having sexual relations with other men seeking healthcare	Qualitative study; Rwanda	Need for respectful and confidential MSM/TGW care training in medical education and healthcare settings; public anti-stigma initiatives highlighted
Ehlers et al. 2001 [13]	To investigate the welfare of gays, lesbians and bisexuals	Questionnaire-based survey; Botswana	Human rights education and advocacy may reduce HIV risk, discrimination, and improve the quality of life for GLB individuals
Muller et al. 2017 [14]	To qualify the availability, accessibility, acceptability and quality of healthcare provisions for LGBT people	Qualitative study; South Africa	Poor clinical competency and inadequate LGBTQ education contributed to dissatisfaction with healthcare services, highlighting the need for accountability and systemic reform
Khalaf et al. 2022 [15]	To explore the attitudes of medical students towards LGBTQ patients	Qualitative study; Malaysia	Limited LGBTQ knowledge among medical students contributes to explicit and implicit bias, reducing healthcare utilisation and highlighting the need for curriculum reform
Torres et al. 2024 [16]	To document the lived healthcare experiences of SGMs	Qualitative study; the Philippines	Accepting healthcare environments improve SGM healthcare access, emphasising the need for respectful SGM training in healthcare curricula
Khatoun et al. 2025 [17]	To assess the healthcare outcomes, experiences and literacy of the LGBTQ community	Cross-sectional study; Lebanon	LGBTQ individuals face higher chronic health burdens and healthcare barriers due to discrimination and financial constraints; positive SOGI self-acceptance improves healthcare access
Commentaries/perspective articles			
Agarwal et al. 2022 [18]	To elucidate the state of LGBT+ healthcare in India	Viewpoint; India	Curriculum pathologises LGBT+ identities; textbooks contain derogatory remarks; need for a reformed medical curriculum to address disparities
Alim et al. 2022 [2]	To document the social stigmatisation and medical violence experienced by intersex individuals in Bangladesh	Perspective; Bangladesh	Intersex individuals are subjected to social and medical violence; lack of treatment and care protocols for intersex children; and non-consensual surgeries on intersex bodies are common
Razai et al. 2023 [19]	To highlight LGBT+ healthcare needs in Afghanistan and call for staff training.	Commentary; Afghanistan	The LGBT+ community is neglected; access to healthcare is limited; discrimination discourages seeking healthcare; need for staff training on LGBT+ health issues
Omohwovo et al. 2026 [20]	To discuss stigma, discrimination, and marginalisation affecting LGBTQ individuals in Africa	Commentary; Africa	LGBTQ individuals face systemic stigma; discrimination in healthcare discourages care seeking; calls for institutional reforms and educational interventions
Web-based contextual sources			
Hindus for Human Rights [21]	To critique recent additions to the Indian medical curriculum and argue that they undermine global medical standards and LGBTQIA+ rights	Blog post; India	The revised curriculum reduced content related to sexual and gender minorities, risk of reinforcing stigma in medical training
Healthcare: Difficulties Of LGBTQ Refugees From The Middle East And North Africa [22]	To explore the challenges faced by LGBTQ refugees, such as a lack of safety and targeted discrimination	Blog post; Switzerland	Accessible healthcare services and trustworthy providers are emphasised to protect the physical and mental health of LGBTQ refugees in the Middle East and North Africa

Discussion

Intersex/DSD Education Gaps in LMICs

**Figure 1:**
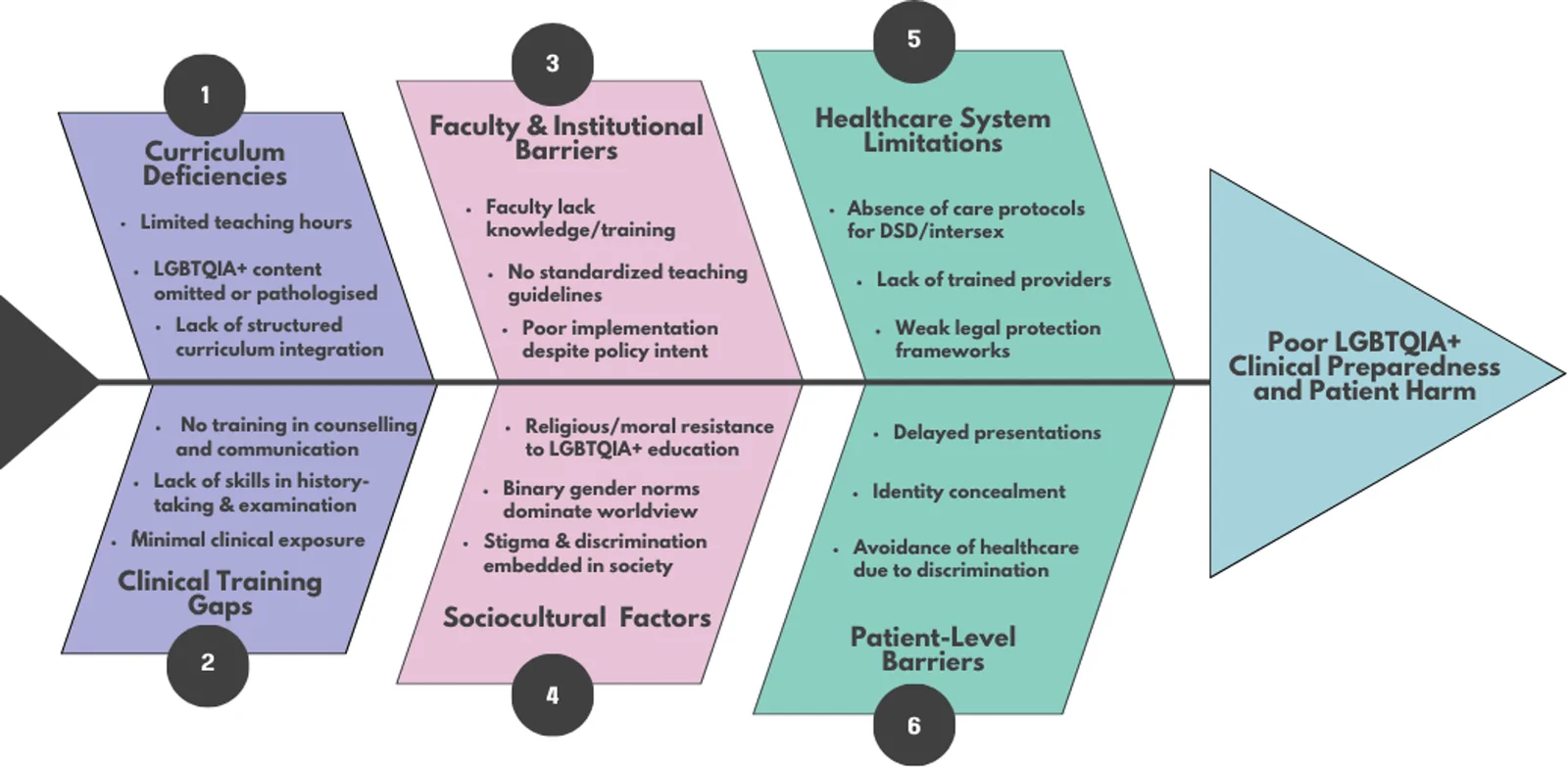
Author-proposed illustration of identified barriers in intersex and LGBTQIA+ education. DSD: differences of sexual development. Made using Canva without AI assistance (Canva Pty Ltd., Sydney, Australia; accessed 2026).

Inadequate knowledge, lack of training, and incompetence emerge as leading factors for the improper healthcare of LGBTQIA+ individuals in LMICs across various continents. Physicians usually view patients through the lens of binary gender norms, i.e., male and female, which hinders their ability to understand issues of intersex individuals [2,9]. This can be interpreted as a major gap in medical education. Since medical students are not sufficiently taught about biologically distinct LGBTQIA+ individuals, they fail to provide the required care in clinical practice. Another example is DSD individuals being advised corrective surgeries and their condition being perceived as a pathology [9]. Such attitudes lead to mistrust between LGBTQIA+ patients and doctors, leading to identity concealment by these patients [11,20]. A comprehensive medical curriculum should address these issues, as knowledge about both surgical and supportive care of intersex individuals is essential to prevent delay or avoidance of healthcare services due to fear of marginalisation, derogatory behaviour, and lack of understanding by HCPs, as reported in literature [23]. The avoidance of healthcare services not only leads to increased incidence of STIs but also increased mental health issues, such as depression, anxiety, risk of suicide, and unhealthy coping mechanisms [24]. A summary of the identified gaps in intersex and LGBTQIA+ education is illustrated in Figure 1.

The incompetence of HCPs in dealing with intersex individuals is largely due to knowledge gaps and the limited time allocated to these conditions in the medical curriculum [9]. Medical curricula worldwide lack content related to understanding and dealing with the problems of DSD individuals [9,18,20]. Studies have shown that medical students, although willing to improve, lack the confidence and competence to deal with intersex health problems [9,18]. Many students demonstrate misunderstandings regarding intersex individuals, as their beliefs are deeply rooted in the dominant dimorphic model of gender and sexuality [2,8,9,18]. These findings underline the need not only to develop academic modules related to intersex and DSD individuals but also to ensure that sufficient time is allocated to these modules. Additionally, theoretical knowledge needs to be supported by clinical practice to maximise efficacy in real-world scenarios. A study conducted among medical students and HCPs in Karachi, Pakistan, revealed that the terms “transgender” and “intersex” were familiar to the majority of the participants; however, several of them did not know the difference between the two [9].

Transgender Health Education Gaps in LMICs

Another study demonstrated that medical students felt unsure about the correct method of obtaining a history and clinically examining transgender patients and, therefore, reported a high perceived need for the inclusion of a trans-health component in the undergraduate medical curriculum. These findings especially support the inclusion of transgender healthcare needs in medical education, as this need is expressed by students themselves. This may reduce the high reliance on relevant specialists, which has resulted in delays in the provision of healthcare for transgender individuals and has made healthcare unaffordable for an already financially vulnerable population [8].

Ethics as a subject is taught to medical students during their clinical years. However, ethics is usually taught within a binary framework of sex, i.e., male or female, and rarely takes into account the existence of intersex and transgender populations. These communities often experience a lack of physician expertise and knowledge regarding basic ethical matters, such as seeking informed consent before performing examinations or procedures. In a study conducted in Turkey, transgender participants reported a lack of positive communication with physicians as a key hurdle to obtaining quality healthcare. Several participants stated that they had to provide repeated clarifications regarding their gender identity and sexual orientation. Participants who could not respond well to these questions clearly failed to establish rapport with physicians and, hence, could not receive the required attention [11].

A study conducted among medical students in Pakistan highlighted insufficient education regarding the health of LGBTQIA+ individuals. However, it reported an overwhelmingly positive response (94%) to incorporating basic knowledge about transgender and intersex individuals into the curriculum [9]. A similar study suggested that students who were exposed to transgender individuals during their preclinical years were able to provide better care to these individuals [8].

Broader LGBTQIA+ Health Education: Sexual Orientation and Other SGM populations

While evidence on curriculum-specific gaps concerning LGBTQI+ individuals in LMICs is limited, several studies document experiences of discrimination, stigma, and inadequate care that reflect deficits in providers’ knowledge. Globally, the LGBTQIA+ community experiences discrimination due to stigma rooted in sociocultural and religious norms and values. Society promotes normative and binary views of sexual orientation, rendering these identities religiously unfaithful or sinful [11]. Political campaigns against them further reinforce anti-LGBTQIA+ ideas and promote the marginalisation of this community [20]. These individuals usually do not attend school, and if they do, they ultimately drop out [10]. Consequently, these individuals are generally unemployed and are unable to meet the costs of interventions or clinical visits [2]. Healthcare professionals are usually not trained in how to attend to such patients and may even refuse to provide care to them based on moral and religious inclinations. As a result, community members either resort to unsafe medical practices or defer their hospital visits. The medical curriculum criminalises such individuals, making it even harder for them to receive basic care. Furthermore, there are no appropriate laws or legislation to protect these individuals. As a result, healthcare has become a privilege for them.

HIC Strategies and Their Applicability to LMICs

Compared with LMICs, healthcare systems in HICs are better equipped to cater for the healthcare needs of LGBTQIA+ individuals. Universities have dedicated sessions on gender identity and LGBTQIA+ terminology, and multiple institutes have incorporated LGBTQIA+ medical education into their curricula, further improving staff capacity (Table 2). Even with these accomplishments, there is still a dire need to improve awareness among medical students in HICs regarding LGBTQ+ individuals. Patients still encounter doctors with inadequate knowledge and unprofessional attitudes. In addition, in countries such as the USA, UK, and Canada, medical students still lack training in addressing the needs of intersex individuals in hospital settings [9]. In LMICs, stigma and social discrimination still pose a huge threat to the well-being of SGM individuals. Students in LMICs have fragmented exposure to these patients because most patients do not visit hospitals to avoid discrimination. The medical curriculum is not designed to equip students to address these issues. Healthcare providers lack knowledge, skills, and expertise regarding these patients [18]. Above all, due to moral and religious inclinations, doctors are usually not interested in acquiring such knowledge and skills. These factors lead to hostile behaviour towards LGBTQIA+ individuals, resulting in poor health outcomes.

**Table 2:** Summary of education strategies and outcomes related to LGBTQI healthcare from high-income countries. TBL: team-based learning, RAT: readiness assurance test, DSD: differences of sexual development, PBL: problem-based learning, AIS: androgen insensitivity syndrome, CBL: case-based learning, PA: physician assistant, SGM: sexual and gender minority, LGBTQI+: lesbian, gay, bisexual, transgender, queer/questioning, intersex, and other sexual/gender minorities. Gupta et al.  [25], Neff et al. [26], Sanchez et al. [27], Salkind et al. [28], Yang et al. [29], Minturn et al. [30]

Study	Country	Participants	Educational Strategy	Key Outcomes
Gupta et al. [25]	USA	36 pediatric residents & medical students	TBL with RAT, case-based application exercises	Improved knowledge and self-efficacy in DSD evaluation and care
Neff et al. [26]	USA	First-year medical students	PBL case; Complete AIS	Improved Knowledge and awareness about DSD; Increased sensitivity to the needs of the LGBTQ community
Sanchez et al. [27]	USA	103 first-year medical students	Single classroom instruction session (pre-post survey)	Significant post-instruction improvements in understanding bisexuality, transgender identity, and LGBTQI+ patient rights
Salkind et al. [28]	UK	Medical students	Compulsory LGBTQ+ teaching module	Increased confidence in LGBTQ+ terminology and clinical assessment skills
Yang et al. [29]	Taiwan	Medical students	Competency-based and game-based teaching related to LGBTQ+ health and medical care	Increased student engagement, participation, and knowledge transmission
Minturn et al. [30]	USA	Preclinical medical students	Structured 10-hour LGBTQ+ health curriculum	Improved clinical confidence and LGBTQ+ terminology knowledge

The experience of HICs in developing and implementing curricula for LGBTQIA+ healthcare offers key insights and strategies that can be adopted in LMICs as well. The use of specific LGBTQIA+ modules in the USA and the UK has notably improved medical students' knowledge and preparedness to provide care to SGMs [30]. While the use of such interventions may be limited in some LMICs due to the massive resources required for overall curriculum reform, shorter and more focused courses can offer many of the same benefits. Minturn et al. demonstrated that even an effectively designed 10-hour course can lead to increased knowledge and clinical confidence among medical students in caring for LGBTQ+ patients [30]. This clearly shows that course design and intentionality play a bigger role than the duration of time allocated to the material. Another challenge in LMICs is the sociocultural stigma associated with the LGBTQI+ population. This severely restricts healthcare access for these individuals, as the content required for their care is not made compulsory in institutions. LGBTQI2SA+ (2S: Two-Spirit) electives and student-run clinics may be the right intervention here, as they offer a voluntary integration pathway for LGBTQI2SA+ content to be taught and learned [31]. A major issue, however, is the preparedness of faculty for teaching such content, as reflected in studies from both HICs and LMICs. 

**Figure 2:**
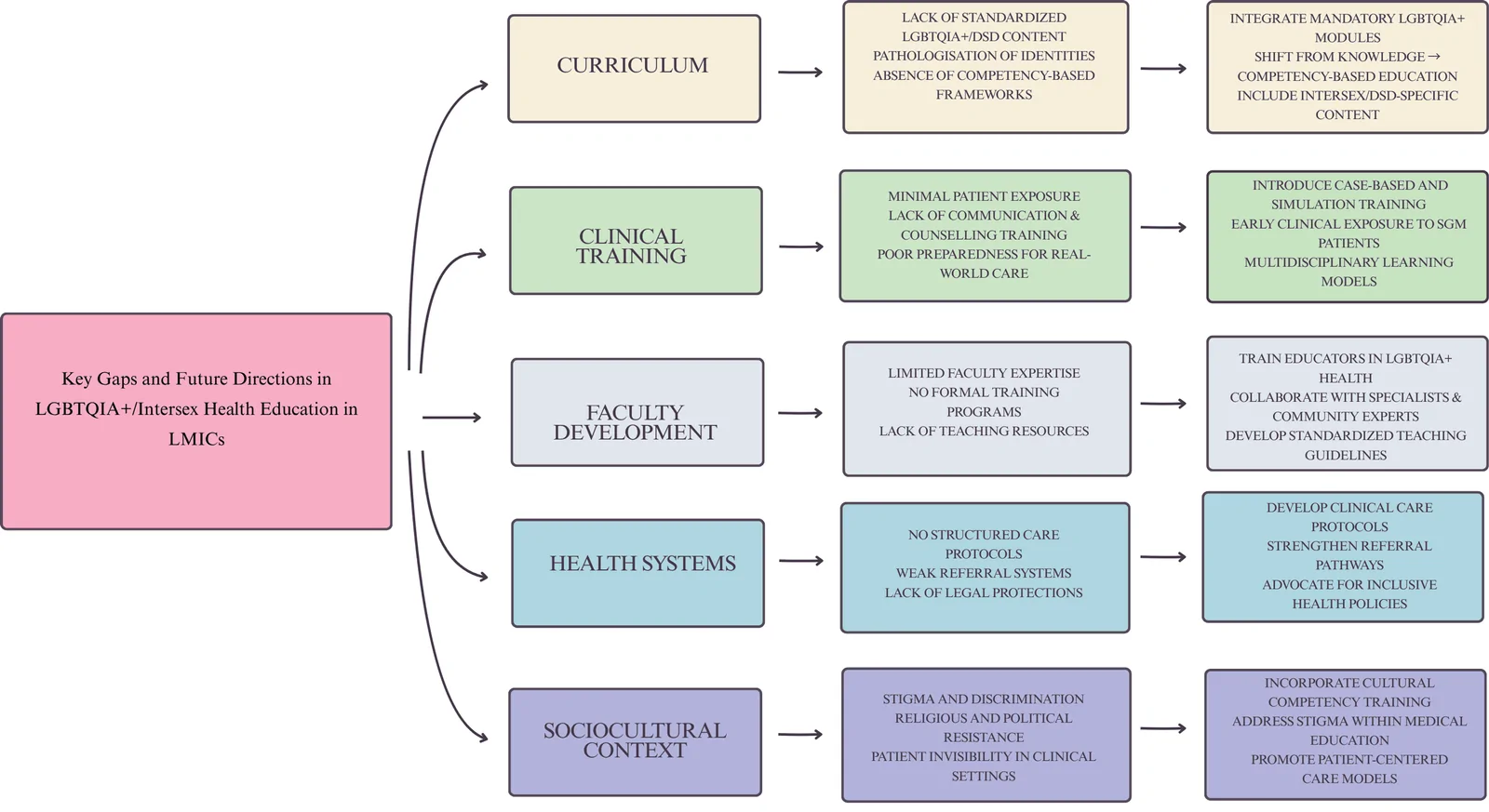
Author-proposed illustration on future directives in intersex and LGBTQIA+ health education. Made using Canva without AI assistance (Canva Pty Ltd., Sydney, Australia; accessed 2026).

The success of such curriculum integration depends on students’ willingness to learn and on faculty members' knowledge and competence. Previous studies have shown that professors had inadequate knowledge regarding transgender individuals; hence, they should educate themselves or collaborate with experts to provide optimum guidance [32]. Implementing case-based learning in LMICs would be timely and economically feasible, as transgender individuals may volunteer for teaching sessions, ultimately improving their care. A summary of the key gaps in LGBTQI+/intersex education in LMICs, along with proposed future directives, has been illustrated in Figure 2. 

Legal and Structural Barriers

Healthcare education and clinical training affect the quality of and access to healthcare for LGBTQI+ individuals; however, these are also affected by the legal infrastructure of the setting in question. In many LMICs, including parts of South Asia, Africa, and the Middle East, same-sex relationships or gender non-conformity are criminalised. This means that despite having the appropriate knowledge and intentions, some physicians may not cater for LGBTQI+ individuals due to fear of social backlash. Further, this leads to the avoidance of seeking healthcare among LGBTQI+ individuals. This highlights the need for broader collaboration among medical educators, public health professionals, and human rights bodies to protect SGMs and improve their health outcomes. 

Limitations

While our findings recognise key patterns and offer insights into the current state of healthcare for LGBTQI+ individuals in LMICs, they are subject to various limitations. The literature included in our review is geographically limited and covers only a few LMICs. This greatly limits the generalisability of our findings. The included literature is also significantly heterogeneous, mainly in terms of study designs. Due to the scarcity of peer-reviewed research on LGBTQIA+, intersex, DSD, and transgender health in low- and middle-income countries (LMICs), this study is limited by the inclusion of grey literature. The quality and methodological rigour of grey literature may vary, although its inclusion expands the body of knowledge. Therefore, it is difficult to collectively synthesise all the findings within this review. While we have included interventional studies from HICs that show improvements in the coverage and effectiveness of curricula for catering to the needs of LGBTQI+ individuals, it remains to be seen whether these can be directly applied to LMICs. Resource constraints, infrastructural weaknesses, and instability can largely limit the application of interventions designed for HICs to the context of LMICs.

## Conclusions

Sexual and gender minorities, LGBTQIA+, transgender, and intersex individuals are subject to substantial health inequities in LMICs. They are vulnerable to the effects of transphobic and stigmatised ideologies, and their healthcare is consistently overlooked in medical education curricula, thus leading to a lack of knowledge and preparedness among medical students and healthcare professionals in catering for the healthcare needs of this population. These factors contribute to reduced health-seeking behaviours in these minority populations, further exacerbating their health conditions. Doctors have an ethical and professional responsibility to provide empathetic and judgement-free care to patients, irrespective of background; therefore, educational reforms are needed. These reforms can include standardised mandatory training in medical schools, as well as elective courses about LGBTQIA+ health, as medical students often show a willingness to learn about their unique health needs and presentations.
